# Wilson’s Presbyterian Almanac

**Published:** 1862-07

**Authors:** John M. Krebs

**Affiliations:** New-York


**WILSON'S PRESBYTERIAN ALMANAC.**—Without solicitation the following is inserted. It embodies our own views of the general and great value of the work, not only to clergymen, but to every well-informed church-member and officer. The price is one dollar and a half a volume. Four are issued. Many a clergyman would like to have the book, but may not see his way clear to spare the price of it. To such we say that we will send either volume free of expense to any clergyman who will send us three dollars for three subscribers to HALL'S JOURNAL OF HEALTH for 1862. We will send the four volumes free of expense for the trouble of obtaining ten subscribers for 1862.

"MESSRS. EDITORS: Some weeks ago I noticed that the *Presbyterian* described *Wilson's Presbyterian Almanac* at some length, and with just commendation. Heartily concurring in this estimate of a work which has now for the fourth time more than justified its prospectus, and is indeed very comprehensive and accurate, I can not but express my regret and surprise that it has not been more extensively encouraged. It is useful to persons of all denominations, but it has especial value for all the various Presbyterian communions. To say nothing of the *ministers*, it would seem as if it ought to be in the hands of *ruling elders* and many *laymen;* and it ought to be suggested to these classes, to avail themselves of the opportunity of possessing the important information it annually collects, with great pains and expense, hitherto inadequately met by the sales. It were to be regretted if, for this want of remunerative sales, the work should cease to be issued.

"Very truly yours, JOHN M. KREBS.

"NEW-YORK, May 8th, 1862."

